# A revealed preference analysis to develop composite scores approximating lung allocation policy in the U.S

**DOI:** 10.1186/s12911-020-01377-7

**Published:** 2021-01-06

**Authors:** Darren E. Stewart, Dallas W. Wood, James B. Alcorn, Erika D. Lease, Michael Hayes, Brett Hauber, Rebecca E. Goff

**Affiliations:** 1 United Network for Organ Sharing, Richmond, VA USA; 2 Research Triangle Institute International, Research Triangle Park, NC USA; 3 Division of Pulmonary, Critical Care, and Sleep Medicine, University of Washington, Washington, USA; 4 RTI Health Solutions, Research Triangle Park, NC USA; 5 University of Washington School of Pharmacy, Seattle, WA USA

**Keywords:** Lung allocation, Organ transplantation, Rank ordered logistic regression, Organ Procurement and Transplantation Network (OPTN), Lung allocation score (LAS), Continuous allocation

## Abstract

**Background:**

The patient ranking process for donor lung allocation in the United States is carried out by a classification-based, computerized algorithm, known as the match system. Experts have suggested that a continuous, points-based allocation framework would better serve waiting list candidates by removing hard boundaries and increasing transparency into the relative importance of factors used to prioritize candidates. We applied discrete choice modeling to match run data to determine the feasibility of approximating current lung allocation policy by one or more composite scores. Our study aimed to demystify the points-based approach to organ allocation policy; quantify the relative importance of factors used in current policy; and provide a viable policy option that adapts the current, classification-based system to the continuous allocation framework.

**Methods:**

Rank ordered logistic regression models were estimated using 6466 match runs for 5913 adult donors and 534 match runs for 488 pediatric donors from 2018. Four primary attributes are used to rank candidates and were included in the models: (1) medical priority, (2) candidate age, (3) candidate’s transplant center proximity to the donor hospital, and (4) blood type compatibility with the donor.

**Results:**

Two composite scores were developed, one for adult and one for pediatric donor allocation. Candidate rankings based on the composite scores were highly correlated with current policy rankings (Kendall’s Tau ~ 0.80, Spearman correlation > 90%), indicating both scores strongly reflect current policy. In both models, candidates are ranked higher if they have higher medical priority, are registered at a transplant center closer to the donor hospital, or have an identical blood type to the donor. Proximity was the most important attribute. Under a points-based scoring system, candidates in further away zones are sometimes ranked higher than more proximal candidates compared to current policy.

**Conclusions:**

Revealed preference analysis of lung allocation match runs produced composite scores that capture the essence of current policy while removing rigid boundaries of the current classification-based system. A carefully crafted, continuous version of lung allocation policy has the potential to make better use of the limited supply of donor lungs in a manner consistent with the priorities of the transplant community.

## Background

Lung allocation decisions in the United States are made according to policies developed by the Organ Procurement and Transplantation Network (OPTN), which is operated by the United Network for Organ Sharing (UNOS) [1]. When a deceased donor lung becomes available, these policies state how potential transplant recipients (candidates) are rank-ordered according to objective characteristics such as donor/candidate blood type compatibility, proximity of the candidate’s transplant hospital to the donor hospital, medical priority, etc. A computerized algorithm, known as the match system, carries out the ranking process by applying discrete policy rules. The available donor lungs are offered first to the top-ranked candidate on the list; if that candidate (or the transplant team) declines the offer, the second-ranked candidate is given the chance to accept, and the process is repeated down the match run waiting list until the lung is accepted for transplant.

Proximity, defined as the distance between the donor hospital and each candidate’s transplant hospital, plays a significant role in prioritizing patients. Proximity is relevant because organ transportation takes time and recovered organs have limited viability outside the body due to the cumulative effects of organ ischemia. Specifically, lung candidates are prioritized according to six concentric circles (zones) around the donor hospital, with zone A encompassing a 250 nautical mile (NM) radius around the donor hospital, zone B between 250 and 500 NM; zone C: > 500 to 1000; zone D: > 1000 to 1500; zone E: > 1500 to 2500; zone F: > 2500 [2]. Regardless of medical priority, candidates in more proximal zones are prioritized ahead of candidates in more distant zones. Within each zone, candidates are further stratified by age brackets—candidates age 12 years or older are prioritized ahead of younger patients for adult donor lungs—and whether or not candidate blood type (ABO) is identical or compatible with the donor.

**Fig. 1:**
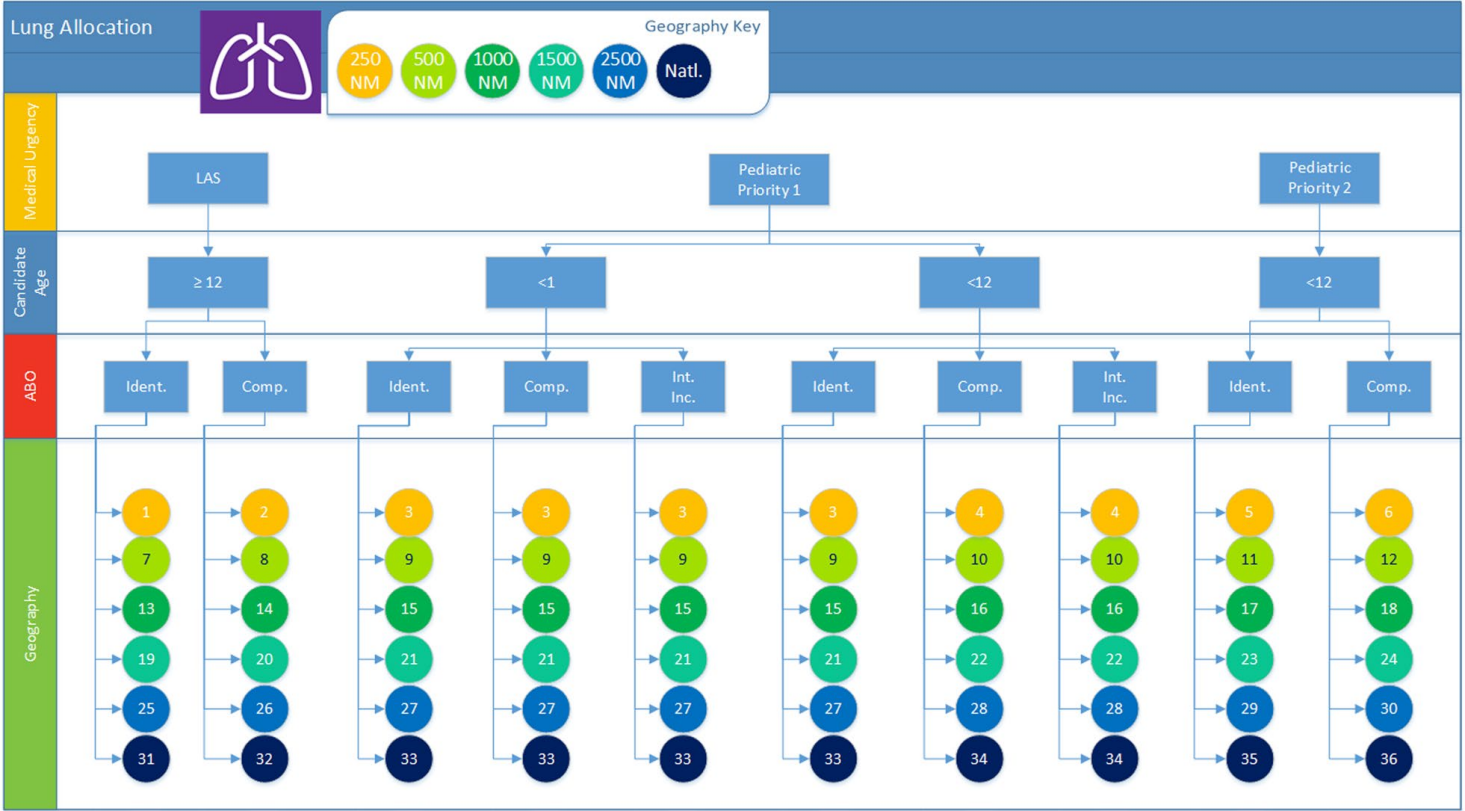
Illustration of allocation of lungs from deceased donors aged at least 18 years old. The chart shows how medical priority, candidate age (younger than 1 year old, younger than 12 years old, and at least 12 years old), ABO (identical, compatible, and incompatible), and proximity define each of the 36 ordered classifications. Within each classification, candidates 12 or older are sorted by (descending) LAS, while younger candidates are sorted by (descending) waiting time. Image created by James Alcorn for the OPTN using Visio 2016

**Fig. 2:**
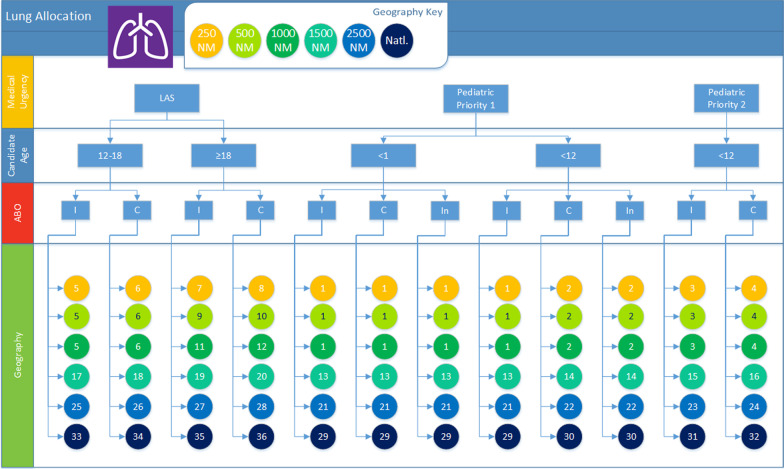
Illustration of allocation of lungs from deceased donors younger than 18 years old. The chart shows how medical priority, candidate age (younger than 1 year old, younger than 12 years old, and at least 12 years old), ABO (identical, compatible, and incompatible), and proximity define each of the 36 ordered classifications. Within each classification, candidates 12 or older are sorted by (descending) LAS, while younger candidates are sorted by (descending) waiting time. Image created by James Alcorn for the OPTN using Visio 2016

This stratification of lung candidates by proximity, blood type compatibility, and age brackets results in 36 ordered “classifications” for the allocation of adult donor lungs, as depicted in Fig. 1. Within each classification, candidates are rank-ordered and prioritized by medical acuity based on descending lung allocation score (LAS) [3] for candidates age 12 or older, and Priority 1 versus 2 status for younger patients. The LAS ranges from 0 to 100 and is composed of two components: the expected 1-year survival time with a transplant, and the expected 1-year survival time without a transplant. In a sense, the LAS measures the patient-specific “net benefit” of lung transplantation. However, since the without-a-transplant (aka, waiting list mortality) component is weighted twice as heavily as the post-transplant survival component, the LAS emphasizes reducing waiting list mortality more so than maximizing post-transplant survival. Figure 2 reveals a similar, 36-classification structure for pediatric lung donor allocation, with two notable differences: children (age < 12 years) having proximity within 1000 NM are prioritized over older candidates, and adolescents (age 12–17) are prioritized ahead of adults (age 18+).

One of the recognized limitations of this classification-driven system is that candidates in a lower classification—even those who are highly medically urgent—are never prioritized ahead of candidates in a higher classification. For example, an LAS 90 candidate 251 NM away is likely severely ill with an elevated mortality risk without a transplant but will be prioritized below a more proximal candidate with a much lower medical priority score (e.g., LAS of 30). Likewise, under the current system, a candidate with a blood type that is identical to the lung donor is always ranked higher than a candidate in the same location with a compatible blood type, even if the latter candidate’s LAS reflects a much greater medical need for transplantation. These types of cases, in which candidates with high medical need are deprioritized due to a strict policy rule or rigid boundary, highlight a significant limitation of the current taxonomic approach to organ allocation.

Although the current approach to organ allocation, in use for decades in the U.S., has helped many thousands in need of life-saving transplants, some experts have wondered whether a continuous, mathematically-derived allocation framework could better align lung allocation policy with requirements of OPTN’s final rule [4] by increasing equity, transparency, and overall allocation efficiency [5, 6]. A mathematical (points-based) system would assign points based on pertinent candidate attributes such as medical urgency (i.e., estimated waiting list mortality without a transplant), expected post-transplant survival time, and factors related to the likelihood of finding a biologically compatible donor, such as blood type. Lung transplant candidates would ultimately be assigned a composite allocation score that would be used to determine their rank ordering on the match run when a donor lung becomes available.

A points-based allocation framework is likely to have at least two major benefits. First, it is more transparent than the current rules-based system because it quantifies how important each candidate attribute is in organ allocation. Second, a points-based allocation framework would allow for the combined effects of many candidate attributes to be considered simultaneously, as opposed to allowing the effect of a single attribute (e.g., blood type identical) to supersede all possible combinations of other attributes.

We performed a study to determine the feasibility of approximating the current lung allocation policy by a mathematically-derived, points-based framework. Feasibility was determined by using data from recent match runs to estimate statistical models that capture the essence of current allocation policies. These models were estimated using discrete choice modeling techniques, which are used extensively in health economics to statistically relate the choices between alternatives made by individuals to the attributes of the alternatives themselves [7, 8]. These models are typically estimated using data collected in experimental settings [9–11]. However, these models can also be estimated using observational data through a revealed preference analysis [12]. The rank-ordering of candidates on each match run explicitly reveals the intrinsic “preferences,” or priorities, embedded in the policy. Our study is unique in that it uses discrete choice modeling to analyze organ allocation preferences generated by a deterministic policy algorithm, as opposed to individual (human) decision-makers.

Our study had three main goals: (1) to demystify the composite score-based (continuous allocation) approach to allocation by showing how current policy can be approximated by a composite score, (2) to quantify the relative importance of factors used in allocation (LAS, distance, blood type, etc.) under current policy, and (3) to provide a viable policy option for implementation of a composite score that adapts the current, classification-based system to the continuous allocation framework.

## Methods

Statistical models were estimated using rank ordered logistic regression [13], a conventional discrete choice modeling technique, applied to rank ordered lists of candidates (“match runs”) generated by the current, OPTN lung allocation policy.

We analyzed all match runs from 2018 (excluding reallocations by an importing organ procurement organization). The year 2018 was chosen to reflect the current allocation lung policy (implemented in November 2017) which is based on geographic concentric circles (zones) [14].

Due to the aforementioned differences in sorting and classification, we developed separate adult and pediatric donor models.

### Adult donor match run data

This study used data from the Organ Procurement and Transplantation Network (OPTN). The OPTN data system includes data on all donors, wait-listed candidates, and transplant recipients in the US, submitted by the members of the OPTN, and has been described elsewhere. The Health Resources and Services Administration (HRSA), US Department of Health and Human Services provides oversight to the activities of the OPTN contractor. IRB exemption was obtained from the US Department of Health and Human Services Health Resources and Services Administration (HRSA).

Data produced by 6466 match runs for 5913 adult lung donors were obtained. An average of 402 candidates were ranked in each match run. As a result, we had data for 2,602,794 ranked candidates, with many candidates appearing on multiple match runs. Candidates screened off of match runs, for example if the donor’s age exceeded the transplant center’s maximum acceptance age, were excluded.

### Pediatric donor match run data

Rankings produced from 534 match runs for 488 pediatric lung donors were used for the pediatric model. An average of 274 candidates were ranked in each match run. As a result, 175,342 observations for estimating the pediatric donor lung allocation model were used.

### Analytic approach to modeling candidate rankings

Analogous to how consumers determine desirability of products based on their attributes, the matching algorithm assigns an unobserved priority score to each candidate during every match run based on that candidate’s characteristics. More formally, the priority score assigned to each candidate j can be represented by the following function:

$${\text{u}}_{{\text{j}}} = {\text{ v}}_{{\text{j}}} + \, \varepsilon_{{\text{j}}} ,{\text{ j }} = { 1}, \ldots ,{\text{ J}},$$

where v_j_ is the observable component of the function that depends on the attributes of the candidate (e.g., location, blood type).

### Adult donor model estimation

The four major attributes used to rank candidates in each match run were included as model covariates: (1) medical priority (LAS), (2) candidate age, (3) candidate’s transplant center proximity to the donor hospital, and (4) blood type identical, compatible, or intended incompatible with the donor. In turn, the observable component of the priority function was specified as follows:

$$\begin{aligned} {\text{V }} = & \, \beta_{{{\text{LAS}}}} \times {\text{ LAS }} + \, \beta_{{{\text{CHILD}}}} \times {\text{CHILD }} + \beta_{{{\text{DISTANCE}}}} \\ & \times {\text{ DISTANCE }} + \, \beta_{{{\text{ABO}}\_{\text{IDENTICAL}}}} \times {\text{ ABO}}\_{\text{IDENTICAL}} \\ \end{aligned}$$

where LAS is a continuous, linear variable that captures the lung allocation score (in our sample, this variable ranges from 0.07 to 96.23); CHILD is a dummy-coded variable that equals 1 for pediatric candidates below the age of 12, and 0 for all other candidates; DISTANCE is a continuous, linear variable that captures the distance from a candidate to the donor hospital in NM (in our sample this variable ranges from 0 to 4415.25 NM); ABO_IDENTICAL is an effects-coded variable that is equal to 1 for candidates with identical blood type as the organ donor and is equal to − 1 for candidates with a compatible (or intended incompatible) blood type to the organ donor.

### Pediatric donor model estimation

The same four attributes were used to rank pediatric donors as adult candidates, with one exception: adolescent (age 12–17) priority was estimated separately from child (0–11) priority to reflect this important distinction in current lung policy. In turn, we specified the observable component of the priority function as follows:

$$\begin{aligned} {\text{V }} = & \, \beta_{{{\text{LAS}}}} \times {\text{ LAS }} + \, \beta_{{{\text{CHILD}}}} \times {\text{CHILD }} + \beta_{{{\text{ADOLESCENT}}}} \times {\text{ADOLESCENT }} \\ & + \beta_{{{\text{DISTANCE}}}} \times {\text{ DISTANCE }} + \, \beta_{{{\text{ABO}}\_{\text{IDENTICAL}}}} \times {\text{ ABO}}\_{\text{IDENTICAL}} \\ \end{aligned}$$

where LAS is a continuous, linear variable that captures the lung allocation score for patients older than 12 years (in our sample, this variable ranges from 0 to 96.23); CHILD is a dummy-coded variable that equals 1 for pediatric candidates below the age of 12, and 0 for all other candidates; ADOLESCENT is a dummy-coded variable that equals 1 for candidates between the ages of 12 and 17 years old and 0 for all other candidates; DISTANCE is a continuous, linear variable that captures the distance from a candidate to the donor hospital in NM (in our sample, this variable ranges from 0 to 4040.68 NM); ABO_IDENTICAL is an effects-coded variable that is equal to 1 for candidates with the same blood type as the donor and is equal to − 1 for candidates with a compatible blood type or incompatible blood type to the donor.

Model estimation was performed using Stata statistical software, Release 16, StatCorp LLC, College Station, TX.

### Determining the relative importance of factors

We used the model coefficients to rank candidate attributes in terms of their relative importance to the ordering of candidates in lung allocation, separately for the adult donor and pediatric donor models. This was done by taking the difference between the score for the most preferred level of an attribute and the score for the least preferred level of the same attribute.

We quantified “exchange rates” to express the relative importance of each factor compared to distance. These rates convey the number of NM required to have the same effect on a candidate’s total score as a change in LAS; blood type identical versus compatible; or pediatric versus adult candidate.

### Evaluating model performance

After estimating the adult and pediatric donor models, we used the resulting parameters to calculate a points-based composite allocation score for each candidate. We used these scores to predict the rank that each of the candidates would have received if the points-based system had been used. The closer these predicted rankings are to the actual rankings, the more the points-based scores reflect the current lung allocation policy. Spearman’s rank correlation coefficient and Kendall’s Tau were used for comparing predicted and actual rankings.

## Results

### Adult donor model results

Table 1 contains the coefficients from the rank-ordered logit model estimated for adult donors. The direction of these coefficients tells us how changing one attribute would change a candidate’s ranking in a given match run. Specifically, we see that candidates are ranked higher if they are adults, have higher LAS scores, are registered at a transplant center closer to the donor hospital, or have an identical blood type to the donor.

**Table 1:** Rank-ordered logit estimates

	Adult donor Mean coefficient (standard error)	Pediatric donor Mean coefficient (standard error)
Medical priority		
Lung allocation score	0.040*** (< 0.001)	0.038*** (< 0.001)
Candidate age		
Less than 12 years old	− 1.601*** (0.026)	1.946*** (0.056)
Between 12 and 17 years old		1.928*** (0.033)
Proximity		
Distance (NM)	− 0.007*** (< 0.001)	− 0.007*** (< 0.001)
Candidate blood type relative to donor blood type		
Identical	1.008*** (< 0.001)	0.978*** (0.004)
Compatible	− 1.008*** (< 0.001)	− 0.978*** (0.004)

(1) Blood type variables are effects coded, candidate age variables are dummy coded, LAS and distance are coded as continuous variables. (2) *** denotes *p* < .01 for statistical significance relative to adjacent categories

Distance in our sample ranges from 0 to 4415.25 NM. This implies that the maximum difference in distance score is 30.907 (30.907 = 0 – (− 0.007 * 4415.25)). By making this calculation for each attribute, we ranked candidate attributes in order of importance, where larger maximum differences imply greater importance. These calculations are presented in Table 2. Based on these calculations, proximity was found to be the most important attribute in lung allocation.

**Table 2:** Ranking candidate attributes by importance in lung allocation

Candidate attribute	Score for most preferred value	Score for least preferred value	Difference	Importance rank
Adult donor model				
Medical priority	3.849	0.000	3.849	2
Candidate age	0.000	− 1.601	1.601	4
Proximity	0.000	− 30.907	30.907	1
Candidate blood type relative to donor blood type	1.008	− 1.008	2.016	3
Pediatric donor model				
Medical priority	3.657	0.000	3.657	2
Candidate age	1.946	0.000	1.946	4
Proximity	0.000	− 28.285	28.285	1
Candidate blood type relative to donor blood type	0.978	− 0.978	1.956	3

Calculations performed using coefficients reported in Table 1, which were rounded to third decimal place

The coefficients were also used to quantify the relative importance of candidate attributes by expressing changes in one attribute in terms of another. For example, as seen in Table 3, reducing a patient’s LAS by 25 points lowers their composite allocation score by exactly 1 point (– 1 = 0.040 * 25). By comparison, increasing the patient’s distance from the donor hospital by 142.857 NM reduces their composite allocation score by exactly 1 point (– 1 = – 0.007 * 142.857). Thus, in terms of the composite score, being 142.857 NM closer to the donor hospital is equivalent to having a 25-point higher LAS. In Table 3, we compared the impact on the composite score of changes in each attribute in terms of changes in a candidate’s proximity to the donor hospital.

**Table 3:** Converting changes in each attribute into changes in NM (“exchange rates”)

Change in attribute	Change in composite allocation score	Equivalent change in NM
Adult donor model		
Medical priority: reduce LAS by 25 points	− 1.000	142.857
Candidate age: reduce candidate age from at least 12 years old to below 12 years old	− 1.601	228.714
Candidate blood type: change candidate blood type from identical to donor to compatible with donor	− 2.016	288.000
Pediatric donor model		
Medical priority: reduce LAS by 25 points	− 0.950	135.714
Candidate age: reduce candidate age from at least 18 years old to below 12 years old	1.946	− 278.000
Candidate blood type: change candidate blood type from identical to donor to compatible with donor	− 1.956	279.429

Calculations performed using coefficients reported in Table 1, which were rounded to the third decimal place

In addition to providing information on the relative importance of individual attributes, we can use the coefficients reported in Table 1 to calculate composite allocation scores for actual or hypothetical candidates. For example, suppose a set of lungs from an adult donor has become available and there are two adult candidates on the match. The first candidate (“A”) is an adult, located 200 NM away from the donor hospital, has a LAS score of 50, and an identical blood type to the donor. The second candidate (“B”) is an adult, located 251 NM away from the donor hospital, has a LAS score of 90, and also an identical blood type to the donor. Based on current policy, the LAS 50 patient would be offered the donor lungs before the much more medically urgent patient with a LAS of 90. However, based on the coefficients in Table 1, the composite score associated with candidate A would be 1.608, and the score associated with candidate B would be 2.851. Therefore, under the composite score approach, the candidate order would be reversed compared to the current, classification-based policy. Despite being outside of the 250 nautical mile boundary, the composite scoring approach would allow the severity of medical need reflected in an LAS of 90 to more than compensate for the relatively minimal additional distance required to ship the organ to this candidate (see Table 4).

**Table 4:** Example of composite score ranking versus current policy ranking for two candidates

Candidate	LAS	Proximity (NM)	Blood type versus donor	Current policy ranking	Composite score	Composite score rank
A	50	200	Identical	1	1.608	2
B	90	251	Identical	2	2.851	1

To assess the degree to which candidate rankings from the composite score reflect rankings under the current policy, we calculated candidates’ scores for 2359 match runs that included at least 10 candidates and quantified the correlation between score-based ranks and current policy ranks. (This comparison is illustrated in the Additional file 1: Table S1 by showing rankings under the current vs. a score-based policy for the first 25 candidates for a sample match run.) We chose to only calculate new rankings for a sample of match runs, because calculating predictive performance metrics is computationally time-consuming when dealing with a large number of observations. Table 5 reports Spearman correlation coefficients and Kendall’s Tau comparing points-based rankings with the actual rankings produced by the matching algorithm for the 2359 match runs. As shown in the table, the mean for both of these coefficients is at least 0.80, suggesting that points-based rankings are (on average) very similar to the actual rankings.

**Table 5:** Predictive performance metrics

	Mean	Minimum	25th percentile	50th percentile	75th percentile	Maximum
Adult donor model (N = 2359)						
Spearman correlation	0.933	0.318	0.916	0.941	0.958	1.000
Kendall’s tau	0.803	0.273	0.765	0.808	0.843	1.000
Pediatric donor model (N = 453)						
Spearman correlation	0.911	0.451	0.897	0.930	0.949	1.000
Kendall’s Tau	0.792	0.551	0.754	0.797	0.833	1.000

We chose only to calculate new rankings for 2359 out of the total 6466 adult donor match runs due to the computationally expensive nature of calculating predictive performance metrics on very large datasets. We only calculated new rankings for 453 of the total 534 pediatric donor match runs because the 81 remaining match runs each included fewer than 10 candidates

**Fig. 3:**
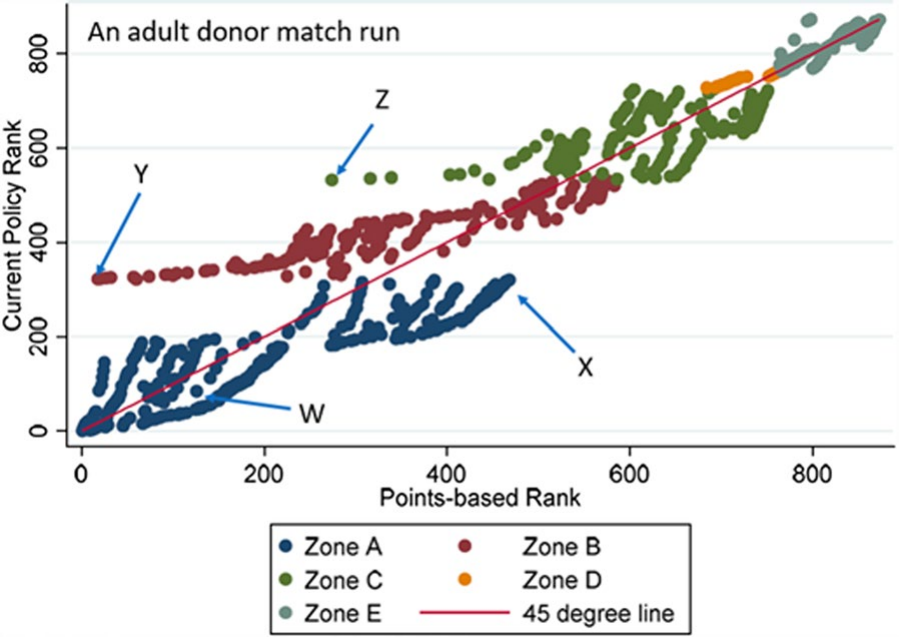
Comparison of actual and predicted rankings for adult donor match run with median Kendall’s Tau. This scatterplot (for one particular adult donor match run) shows that candidate rankings under a composite-score based approach are generally highly correlated with those under current policy. However, the figure also reveals important ways in which the score-based approach rank orders patients differently than current policy by eliminating hard boundaries. Four candidate profiles are shown to illustrate salient differences in rankings: Candidate W: LAS (36), distance (201.1), ABO(O), Adult. Candidate X: LAS (32), distance (222.9), ABO(A), Adult. Candidate Y: LAS (86), distance (275.7), ABO(O), Adult. Candidate Z: LAS (74), distance (528.6), ABO(O), Adult. Image created by Dallas Wood using Stata Version 16

Figure 3 illustrates a scatter plot of the current policy rankings and points-based rankings for an adult donor match run with 873 candidates and having the median Kendall’s Tau of 0.808. If the current policy rankings and points-based rankings were identical, all points on this scatter plot would lie on the 45°-line extending from the origin (illustrated in red). In reality, we see that though the rank correlation is high, there are still notable differences between the two sets of rankings. Specifically, some candidates in zones B and C—for example, candidates Y and Z as annotated on the figure—have higher priority (numerically lower ranking) under the points-based system than under current policy. This is because the current system grants absolute priority to candidates in more proximal zones. By contrast, under a points-based system, candidates farther away from a donor hospital may have other attributes (e.g. higher LAS scores) that overcome their lack of proximity.

### Pediatric donor model results

Table 1 contains the coefficients from the rank-ordered logit model estimated from pediatric donor match runs. As in the adult model, these coefficients were used to make inferences about how candidate attributes influence donor lung allocation. Specifically, the score-based system ranks candidates higher if they are younger than 12 years old, have a higher LAS, are registered at a transplant center closer to the donor hospital, or have identical blood type to donors. Based on calculations shown in Table 2, proximity was found to be the most important attribute in allocating pediatric donor lungs.

Table 3 shows that for the pediatric donor model, an increase in 25 LAS points is equivalent to being 135.714 NM closer in terms of the composite score. Table 5 reports Spearman correlation coefficients (mean of 0.911) and Kendall’s Tau (0.792) for comparing points-based rankings with the actual rankings produced by the matching algorithm for all 453 pediatric donor match runs having at least 10 candidates.

**Fig. 4:**
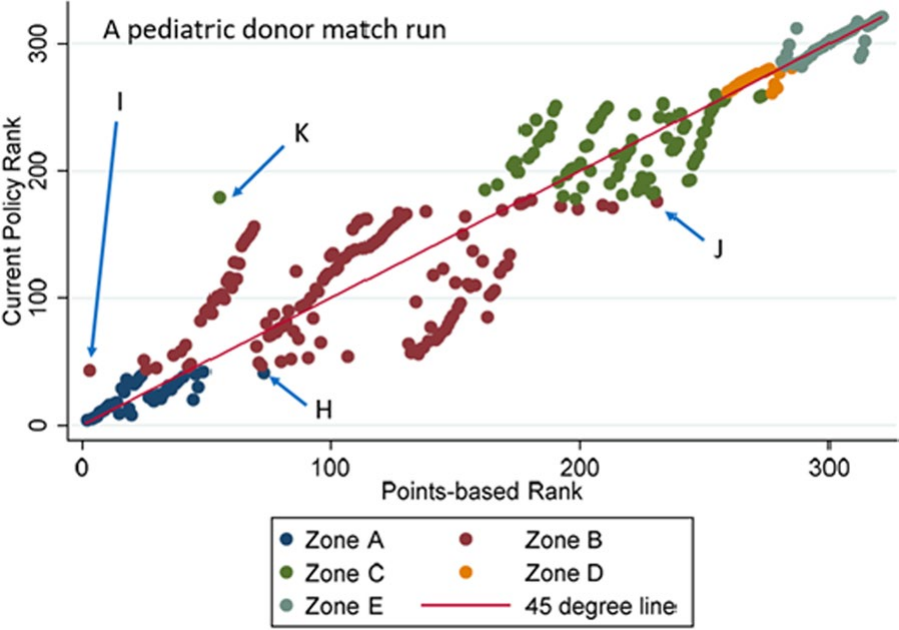
Comparison of actual and predicted rankings for pediatric donor match run with median Kendall’s Tau. This scatterplot (for one particular pediatric donor match run) shows that candidate rankings under a composite-score based approach are generally highly correlated with those under current policy. However, the figure also reveals important ways in which the score-based approach rank orders patients differently than current policy by eliminating hard boundaries. Four candidate profiles are shown to illustrate salient differences in rankings: Candidate H: LAS (69), distance (190.5), ABO(AB), Adult. Candidate I: LAS (92), distance (314.3), ABO(A), Adult. Candidate J: LAS (33), distance (484.1), ABO(AB), Adult. Candidate K: LAS (86), distance (523.2), ABO(A), Adult. Image created by Dallas Wood using Stata Version 16

Figure 4 illustrates a scatter plot of the current policy rankings and points-based rankings for a 138-candidate, pediatric donor match run having the median Kendall’s Tau of 0.797. As in the adult donor model, we see that there are some differences between the two sets of rankings. Specifically, as seen with the adult donor model, candidates in further away zones are sometimes ranked higher than more proximal candidates under the points-based system compared to current policy. For example, though all zone A candidates would rank ahead of Candidate I under the classification-based system, Candidate I would rank near the very top under a points-based system due to having an extremely high LAS of 92.

## Conclusions

Although the computerized match system plays a critical role in matching donor organs and candidates, the value judgments inherent in the current classification-based system can be opaque. An alternative way to make organ allocation decisions is to leverage a points-based framework that transparently expresses the relative importance of proximity, medical priority, and other factors to form a mathematically-derived, composite score.

Our analysis sought to determine if preferences and priorities within current lung allocation policy could be captured, at least approximately, by composite scores. First, we used rank ordered logistic regression, a conventional discrete choice modeling technique, to estimate two statistical models based on match runs from 2018—one for adult donor lungs and one for pediatric donor lungs. These statistical models estimated scores that quantified how important the following candidate attributes are in lung allocation rankings: (1) medical priority (i.e., LAS), (2) candidate age, (3) candidate proximity to donor hospital, and (4) blood type. Second, we confirmed that the estimated scores approximately reflect the current lung allocation policy by comparing score-based candidate rankings with rankings from the current system. Overall, we demonstrate that these rankings are highly correlated with the original ranks produced by the matching algorithm.

The proximity of the candidate’s transplant hospital to the donor hospital was found to be the most important factor in a composite score that reflects the current policy. In terms of attribute “exchange rates,” 25 LAS points equates to just 143 NM, implying that a nearby candidate with LAS of 45 would be prioritized ahead of a LAS 70 candidate just 150 NM further away. The rationale for prioritizing patients based on proximity reflects both system efficiency and organ viability considerations, as transporting lungs over long distances incurs transportation costs, travel time by the surgical recovery team, and potentially detrimental effects of organ ischemia time [15–19]. The manner and degree to which proximity should influence candidate rankings is a matter of ongoing debate [20–22].

Although the results we present are insightful, it is important to note that they are subject to limitations. First, due to the opacity of the current, classification-based system, the precise value judgments that manifested from the revealed preference analysis do not necessarily reflect policymakers’ intended value judgments. Second, the model specification we used for several key attributes oversimplified the way these attributes entered the lung allocation rankings. For example, in both the adult donor and pediatric donor models, we only estimated a single coefficient for candidates younger than 12 years old. As a result, we did not differentiate between candidates with “Priority 1” from candidates with “Priority 2” [1] status, which may slightly reduce the accuracy of both models’ predictions. We also simplified the composite score by omitting the waiting time attribute, which plays a subordinate role in lung allocation (essentially serving merely as a tiebreaker between two candidates with identical LAS or medical priority).

**Fig. 5:**
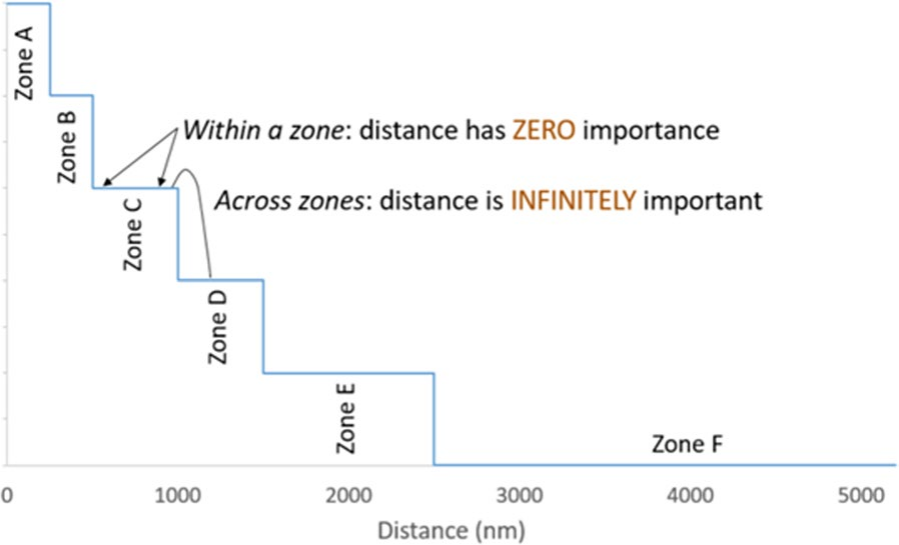
Illustration of the importance of distance in current policy. In the current lung allocation system in which classifications are defined, in part, by geographic zones representing concentric circles around the donor hospital, the role of proximity in candidate rank-ordering varies: within a zone, proximity has zero importance, but since candidates in further-away zones cannot supersede candidates in a more proximal zone, proximity effectively has infinite importance across zones. Image created by Darren Stewart using Microsoft Excel Version 2016

**Fig. 6:**
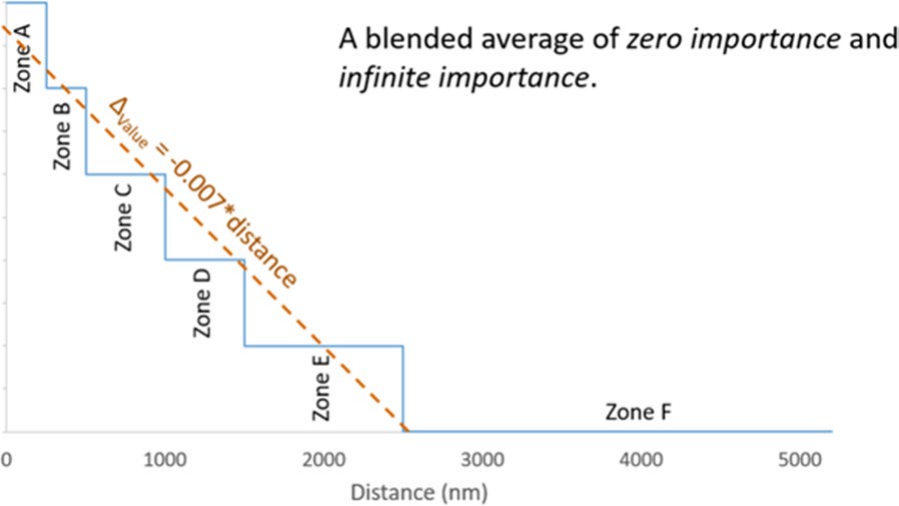
Illustration showing how revealed preference distance effect is a *blended estimate*. A linear relationship between distance and candidate priority was assumed; this was an intentional oversimplification to aid model interpretability and reflect the spirit of the continuous distribution framework, in which incremental changes in numerical factors such as distance are to contribute incrementally to the composite score. The − 0.007 coefficient estimated for both the adult and pediatric donor models can be thought of as a blended average of the current relationship between distance and priority, which varies between zero importance (within zones) to infinite importance (across zones). Image created by Darren Stewart using Microsoft Excel Version 2016

In the current policy, distance is either infinitely important (across zones) or of zero importance (within zones). This composite scoring approach yields an average estimate of the impact of distance as a continuous linear function (Figs. 5, 6). Though specification of distance as a continuous, linear term instead of a zone-based categorical variable departs from the structure of current policy, this linear parameterization is more consistent with the spirit and intent of composite-score based allocation.

Revealed preference analysis of match runs produced a composite score that captures the essence of current policy while removing hard boundaries. As highlighted in Table 4, this approach avoids artificial boundaries that currently preclude a candidate with a greater medical priority (LAS) from being ranked higher than a lower-LAS patient solely because the higher-LAS candidate’s transplant hospital is on the other side of a geographic zone. The linear parameterization also permits highly interpretable value judgment expressions (i.e., “exchange rates”), as shown in Table 3.

So, could developing composite scores through revealed preference analysis be the solution to migrating lung allocation policy to the continuous allocation framework? This is a possibility, although recent policy deliberations of the OPTN Lung Transplantation Committee (Lung Committee) have suggested the need for the new system to include several new attributes—for example, candidate height and degree of Human Leukocyte Antigen (HLA) allo-antibody sensitization—that are not included in current policy. These factors would somehow need to be appended to the composite scores shown here. An alternative approach would be to develop an entirely new composite scoring system based on a reevaluation of the degree to which proximity and other factors should be valued relative to medical need, as opposed to deriving the composite score from the current policy, which some have criticized [23].

The primary value in these revealed preference-derived scores, we believe, is in highlighting the degree to which each of the four key attributes influences candidate rank-ordering under the current policy for comparison to an idealized policy (i.e., the relative importance the OPTN and broader transplant community believe these factors *should* have in a new allocation system).

The Lung Committee is exploring the use of analytic hierarchy process (AHP), a structured approach to eliciting value judgments and preferences from stakeholders, to establish this idealized policy [24–28]. The AHP results have been compared with the revealed preference analysis presented herein to stimulate discussion on the appropriate level of importance to be placed on each attribute, in accordance with federal regulation governing organ allocation policies [4].

In theory, a carefully crafted, continuous version of lung allocation policy has the potential to make greater use of the limited supply of donor lungs by transplanting more patients with the highest predicted benefit of transplant while also ensuring that access to lungs is equitable and accounting for inefficiencies related to transportation logistics over long distances and under tight time restrictions. Simulation modeling will be used to forecast the impact of composite scoring options compared to current policy, and as with all OPTN policy changes, the effects of the new policy on patients and the transplant network as a whole will be closely monitored to determine if adjustments are necessary. Policy changes will entail fine-tuning the score (e.g., increasing the coefficient of some variables and decreasing others) as opposed to shuffling classifications. The composite scoring approach should allow lung allocation to readily adapt to future innovations; for example, if technologies such as ex-vivo lung perfusion [29] become widely used and can reduce the deleterious effect of organ ischemia time associated with travel, the score can be tuned by reducing the relative importance of proximity compared to other factors. And as evidence supporting an association between other factors (e.g., donor/candidate size-matching; use of extra-corporeal membrane oxygenation) and recipient outcomes is generated, the score can be augmented to account for such discoveries. This continuous composite score approach to lung allocation policy has the potential to more effectively utilize the limited supply of donor lungs in a manner consistent with the priorities and preferences of the transplant community.

## Supplementary Information

**Additional file 1.** Candidates are sorted by current allocation policy rank. Candidates’ composite score ranks, derived through revealed preference logistic regression analysis, are shown for comparison. Some candidates' position on the match run would improve under the composite scoring, whereas other candidates would appear further down on the match run.

## Data Availability

Deidentified OPTN match run data were made available to Research Triangle Institute (RTI) under the terms of a data use agreement signed and returned to UNOS. The data that support the findings of this study are available from the OPTN but restrictions apply to the availability of these data, which were used under license for the current study. Data are available upon reasonable request from the OPTN.

## Abbreviations

AHP: Analytic hierarchy process
DSA: Donor service area
IRB: Institutional Review Board
HRSA: Health Resources and Services Administration
LAS: Lung allocation score
NM: Nautical mile
OPTN: Organ Procurement and Transplantation Network
UNOS: United Network for Organ Sharing
