# Supplementary material for: A revealed preference analysis to develop composite scores approximating lung allocation policy in the U.S
Source: BMC Med Inform Decis Mak. 2021 Jan 6;21:8. doi: 10.1186/s12911-020-01377-7 (PMC7789710; doi:10.1186/s12911-020-01377-7)
Supplement: Supplementary file 1 — Additional file 1. Candidates are sorted by current allocation policy rank. Candidates’ composite score ranks, derived through revealed preference logistic regression analysis, are shown for comparison. Some candidates' position on the match run would improve under the composite scoring, whereas other candidates would appear further down on the match run. [file 12911_2020_1377_MOESM1_ESM.docx]

**Table S1.** First 25 candidates ranked in sample adult donor match run (Kendall’s Tau .95).

| **Current Lung Allocation Policy Rank** | **Composite Allocation Score Rank** | **Composite Allocation Score** | **LAS** | **Candidate Age** | **Zone** | **Distance** | **Blood Type** |
| --- | --- | --- | --- | --- | --- | --- | --- |
| 1 | 1 | 1.529 | 54.685 | 44 | A | 225.93 | Identical |
| 2 | 2 | 0.812 | 36.651 | 68 | A | 225.93 | Identical |
| 3 | 3 | 0.715 | 34.223 | 43 | A | 225.93 | Identical |
| 4 | 4 | 0.702 | 33.896 | 34 | A | 225.93 | Identical |
| 5 | 5 | 0.675 | 33.212 | 64 | A | 225.93 | Identical |
| 6 | 6 | 0.664 | 32.927 | 61 | A | 225.93 | Identical |
| 7 | 7 | -0.674 | 50.000 | 29 | A | 225.93 | Compatible |
| 8 | 10.5 | -0.912 | 44.008 | 56 | A | 225.93 | Compatible |
| 9 | 10.5 | -0.912 | 44.008 | 56 | A | 225.93 | Compatible |
| 10 | 15 | -1.060 | 40.306 | 48 | A | 225.93 | Compatible |
| 11 | 21 | -1.294 | 34.415 | 38 | A | 225.93 | Compatible |
| 12 | 8 | -0.699 | 55.445 | 66 | C | 534.46 | Identical |
| 13 | 9 | -0.885 | 50.788 | 69 | C | 534.46 | Identical |
| 14 | 12 | -0.945 | 49.278 | 57 | C | 534.46 | Identical |
| 15 | 13 | -0.981 | 48.366 | 47 | C | 534.46 | Identical |
| 16 | 14 | -1.035 | 47.002 | 60 | C | 534.46 | Identical |
| 17 | 16 | -1.157 | 43.938 | 43 | C | 534.46 | Identical |
| 18 | 17 | -1.222 | 42.288 | 29 | C | 534.46 | Identical |
| 19 | 18 | -1.231 | 42.065 | 61 | C | 534.46 | Identical |
| 20 | 19 | -1.238 | 41.902 | 52 | C | 534.46 | Identical |
| 21 | 20 | -1.256 | 41.438 | 66 | C | 534.46 | Identical |
| 22 | 23 | -1.343 | 39.269 | 53 | C | 534.46 | Identical |
| 23 | 24 | -1.371 | 38.546 | 62 | C | 534.46 | Identical |
| 24 | 25 | -1.421 | 37.299 | 67 | C | 534.46 | Identical |
| 25 | 26 | -1.481 | 35.785 | 62 | C | 534.46 | Identical |
